# Hyperthermia ablation combined with transarterial chemoembolization versus monotherapy for hepatocellular carcinoma: A systematic review and meta‐analysis

**DOI:** 10.1002/cam4.4350

**Published:** 2021-10-16

**Authors:** Zheng Li, Qiang Li, Xiaohu Wang, Weiqiang Chen, Xiaodong Jin, Xinguo Liu, Fei Ye, Zhongying Dai, Xiaogang Zheng, Ping Li, Chao Sun, Xiongxiong Liu, Qiuning Zhang, Hongtao Luo, Ruifeng Liu

**Affiliations:** ^1^ Institute of Modern Physics Chinese Academy of Sciences Lanzhou China; ^2^ Key Laboratory of Heavy Ion Radiation Biology and Medicine of Chinese Academy of Sciences Lanzhou China; ^3^ Gansu Provincial Key Laboratory of Basic Research on Heavy Ion Radiation Application in Medicine Lanzhou China; ^4^ Lanzhou Heavy Ion Hospital Lanzhou China; ^5^ University of Chinese Academy of Sciences Beijing China

**Keywords:** COVID‐19, hepatocellular carcinoma, hyperthermia ablation, meta‐analysis, SARS‐CoV‐2, transarterial chemoembolization

## Abstract

**Background and aims:**

The existing evidence has indicated that hyperthermia ablation (HA) and HA combined with transarterial chemoembolization (HATACE) are the optimal alternative to surgical resection for patients with hepatocellular carcinoma (HCC) in the COVID‐19 crisis. However, the evidence for decision‐making is lacking in terms of comparison between HA and HATACE. Herein, a comprehensive evaluation was performed to compare the efficacy and safety of HATACE with monotherapy.

**Materials and Methods:**

Worldwide studies were collected to evaluate the HATACE regimen for HCC due to the practical need for global extrapolation of applicative population. Meta‐analyses were performed using the RevMan 5.3 software (The Nordic Cochrane Centre, The Cochrane Collaboration, Copenhagen, Denmark).

**Results:**

Thirty‐six studies involving a large sample of 5036 patients were included finally. Compared with HA alone, HATACE produced the advantage of 5‐year overall survival (OS) rate (OR:1.90; 95%CI:1.46,2.46; *p *< 0.05) without increasing toxicity (*p *≥ 0.05). Compared with TACE alone, HATACE was associated with superior 5‐year OS rate (OR:3.54; 95%CI:1.96,6.37; *p *< 0.05) and significantly reduced the incidences of severe liver damage (OR:0.32; 95%CI:0.11,0.96; *p *< 0.05) and ascites (OR:0.42; 95%CI:0.20,0.88; *p *< 0.05). Subgroup analysis results of small (≤3 cm) HCC revealed that there were no significant differences between the HATACE group and HA monotherapy group in regard to the OS rates (*p* ≥ 0.05).

**Conclusions:**

Compared with TACE alone, HATACE was more effective and safe for HCC. Compared with HA alone, HATACE was more effective for non‐small‐sized (>3 cm) HCC with comparable safety. However, the survival benefit of adjuvant TACE in HATACE regimen was not found for the patients with small (≤3 cm) HCC.

## Graphical abstract

In this study, we have demonstrated that HATACE for HCC is superior to TACE monotherapy with respect to either efficacy or safety. HATACE is more effective than HA monotherapy with comparable safety for non‐small‐sized (>3 cm) HCC. Compared with HATACE, HA monotherapy could provide comparable survival benefit for the patients with small (≤3 cm) HCC. Namely, adjuvant TACE is not necessary for HA therapy in treating small HCC. Although there are some deficiencies as discussed in the limitations section, this research could provide a comprehensive reference for clinical decision‐making on the base of the 36 included studies and the adequately large sample size of 5036 patients. In addition, we should pay more attention to HA and HATACE due to their superiorities in the SAUCCC.

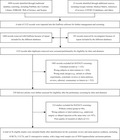

## 1 INTRODUCTION

The coronavirus disease 2019 (COVID‐19), an infectious disease caused by a novel coronavirus named severe acute respiratory syndrome coronavirus 2 (SARS‐CoV‐2),^1^, ^2^ was declared as a global pandemic by the World Health Organization (WHO) on 11 March 2020.^3^ As of 2 September 2021, there have been 218,205,951 confirmed cases of COVID‐19, including 4,526,583 deaths, according to the global data reported to WHO from almost all countries and regions.^4^ The COVID‐19 pandemic has tremendously altered routine medical service provision worldwide and imposed unprecedented challenges to the global healthcare systems.^2^, ^3^, ^4^, ^5^, ^6^, ^7^ There exists intricate relationship among COVID‐19, cancer, and its treatment.^8^, ^9^, ^10^, ^11^, ^12^, ^13^, ^14^ The radical transformation of cancer management caused by COVID‐19 has deeply affected the patients with hepatocellular carcinoma (HCC) in the specific areas undergoing the uncontrollable COVID‐19 crisis (SAUCCC).^6^, ^15^ Many HCC patients without COVID‐19 cannot get normal surgical resection (SR) because of the high risk of SARS‐CoV‐2 infection after SR in the SAUCCC.^8^, ^9^, ^10^, ^11^, ^12^, ^13^, ^14^ But on the other hand, giving up SR or delay in SR, a compromise strategy occurring because of the COVID‐19 pandemic, has immensely increased the risk of malignant death.^4^, ^15^, ^16^ What is the solution for this dilemmatic predicament widespread in the SAUCCC? This dilemma could be settled easily and perfectly if there is an idealized therapy modality that only kills cancer cells without any toxicity for normal tissue.^8^, ^9^, ^10^, ^11^, ^12^, ^13^, ^14^ Therefore, the optimization of therapeutic safety is the realistic and feasible solution for the predicament of HCC treatment during the COVID‐19 crisis.^8^, ^9^, ^10^, ^11^, ^12^, ^13^, ^14^

Minimal invasiveness has become a crucial principle for HCC treatment in the SAUCCC.^8^, ^9^, ^10^, ^11^, ^12^, ^13^, ^14^ Namely, the weight of therapeutic safety is amplified due to SARS‐CoV‐2.^8^, ^9^, ^10^, ^11^, ^12^, ^13^, ^14^ Several meta‐analyses^17^, ^18^, ^19^, ^20^, ^21^ revealed that micro‐invasive hyperthermia ablation (HA) or HA combined with transarterial chemoembolization (HATACE) could be effective alternative to SR for applicable HCC patients with added benefit of lower morbidity of adverse effects and complications. Compared with SR,^17^, ^18^, ^19^, ^20^, ^21^ HA (or HATACE) is associated with lower incidence of complications, less intraoperative blood loss, shorter operative time, and shorter length of hospitalization stay, which is significant to reduce the risk of SARS‐CoV‐2 infection in the SAUCCC.^8^, ^9^, ^10^, ^11^, ^12^, ^13^, ^14^ Therefore, both HA and HATACE possess unique superiorities among multifarious therapies for applicable HCC patients in the SAUCCC.^17^, ^18^, ^19^, ^20^, ^21^, ^22^, ^23^, ^24^, ^25^, ^26^, ^27^, ^28^, ^29^, ^30^, ^31^, ^32^, ^33^, ^34^, ^35^, ^36^, ^37^ However, the evidence for decision‐making is lacking in terms of comparisons between HATACE and HA monotherapy for HCC patients. Accordingly, we carried out this systematic review and meta‐analysis to comprehensively compare the efficacy and safety of HATACE with HA or TACE monotherapy for treating HCC patients.

## 2 MATERIALS & METHODS

A pre‐retrieval procedure was implemented to ensure that the best results of literature retrieval could be obtained, which started on 11 March 2020. A preliminary and rapid systematic review was conducted before this study to ascertain how to design this study scientifically and accurately. Systematic review and meta‐analysis were identified as the preferred research method for this study due to the actual need of comprehensive and worldwide data for global extrapolation of applicative population. No ethical approval or patient consent was required for the systematic review and meta‐analysis as the data originated from previously published studies.

### 2.1 Inclusion and exclusion criteria of study selection

Studies were included if they matched the following criteria based on the pilot study of systematic review and meta‐analysis. (i) Participants: Patients were diagnosed with primary HCC by histopathology and imageological examination, while the patients with metastatic liver cancer were excluded. (ii) Intervention and comparison: HA modalities included radiofrequency ablation (RFA) and microwave ablation (MWA) in this article. Studies for HATACE should compare HATACE with monotherapy of HA (RFA/MWA) or TACE. (iii) Outcomes: Outcomes of evaluation were including overall survival (OS), adverse effects, and complications. The primary endpoint was OS as conventional assessment criteria, which is defined as the time from random assignment to the last follow‐up or death. (iv) Study type: Studies with control group were included to compare HATACE with monotherapy of HA or TACE for HCC, such as randomized controlled trial (RCT), controlled clinical trial (CCT), and propensity score matching study (PSMS). Different criteria of study type were performed for general meta‐analysis and sensitivity analysis due to different aims.

Publications were excluded if they were (i) retraction by published journals; (ii) duplicate publications; (iii) clinical research without control group; and (iv) inappropriate article type including cellular or animal experiments, letters, editorials, commentaries, protocols, reviews, systematic reviews or meta‐analyses.

### 2.2 Search strategy and study screening

The pre‐retrieval was performed on 11 March 2020, and the comprehensive retrieval was started on 15 April 2020, following the pilot systematic review. The retrieval was updated every month during the research process in order to acquire the latest data of reports. The final retrieval time was 15 May 2021. We searched five international databases including the Cochrane Library, Web of Science, PubMed, Embase, and Scopus. We also searched other supplementary resources, such as the Google Scholar, Medical Matrix, reference lists of relevant reviews and included papers, COVID‐19 Open Research Dataset Challenge (CORD‐19), COVID‐19 Research Database (WHO), and WHO International Clinical Trials Registry Platform. No restrictions were set for study language, publication date, and publication status. Additionally, we also communicated with some colleagues to identify the potential unpublished trials for avoiding publication bias. Studies were selected according to the inclusion and exclusion criteria through two stages: the first stage was evaluation of titles and abstracts, followed by full­text review as the second stage.

### 2.3 Data extraction and data analysis

Data were extracted from each included article using standardized forms. Meta‐analysis should not count overlapping populations in any outcome synthesis to avoid the bias of data double counting. Therefore, when multiple publications from the same institution were identified as duplicates (e.g., studies reporting the same series of patients at different phases or different perspectives), we chose the most recent updated papers with the largest sample size or longest follow‐up duration for the quantitative synthesis of the meta‐analyses.

The meta‐analyses were performed with the RevMan version 5.3 software (The Nordic Cochrane Centre, The Cochrane Collaboration) provided by the Cochrane organization. Odds ratios (ORs) and 95% confidence intervals (CIs) were used as the summary statistics for dichotomous data, which were calculated using the statistical method of Mantel–Haenszel and the analysis model of fixed‐effect or random‐effects according to the estimate of heterogeneity. The two‐sided level of statistical significance was denoted as the two‐tailed *p* value below the threshold of 0.05. The statistical heterogeneity (or consistency) among studies was measured with the Cochran's Q χ² test and I² test. A *p* value of up to 0.10 was considered significant heterogeneity in the Cochran's Q χ² test. An I² value of 0% indicates the optimal consistency (or no observed heterogeneity), and larger values indicate the increasing heterogeneity (or decreasing consistency). I² statistic ≤50% was considered indicative of low heterogeneity with the fixed‐effect model, and >50% was considered indicative of high heterogeneity with the random‐effects model.

### 2.4 Sensitivity analysis and subgroup analysis

Randomized controlled trials (RCTs) and systematic reviews of RCTs could provide the most reliable evidence about the effects of healthcare interventions.^38^ Therefore, we chose RCTs for sensitivity analysis to test the robustness of our findings from the meta‐analyses. Only RCTs were included in the sensitivity analysis in order to avoid potential biases associated with case ascertainment or others from non‐RCT studies. Risk ratios (RRs, instead of ORs) and 95% CIs were used as the summary statistics for dichotomous data in the sensitivity analyses of RCTs, while the others were the same as above. The risk of bias in RCTs was assessed in both table and figure formats according to the Cochrane Collaboration's tool for randomized trials.^38^

There is conflicting debate as to whether it is necessary to implement additional TACE of combination therapy for small (≤3 cm) HCC. Accordingly, HCC was classified into two grades according to tumor size: small‐sized HCC (diameter of 3 cm or less) and non‐small‐sized HCC (diameter greater than 3 cm). Subgroup analysis was implemented on the basis of the size classification.

## 3 RESULTS

### 3.1 Results of the systematic review and meta‐analysis

**FIGURE 1:**
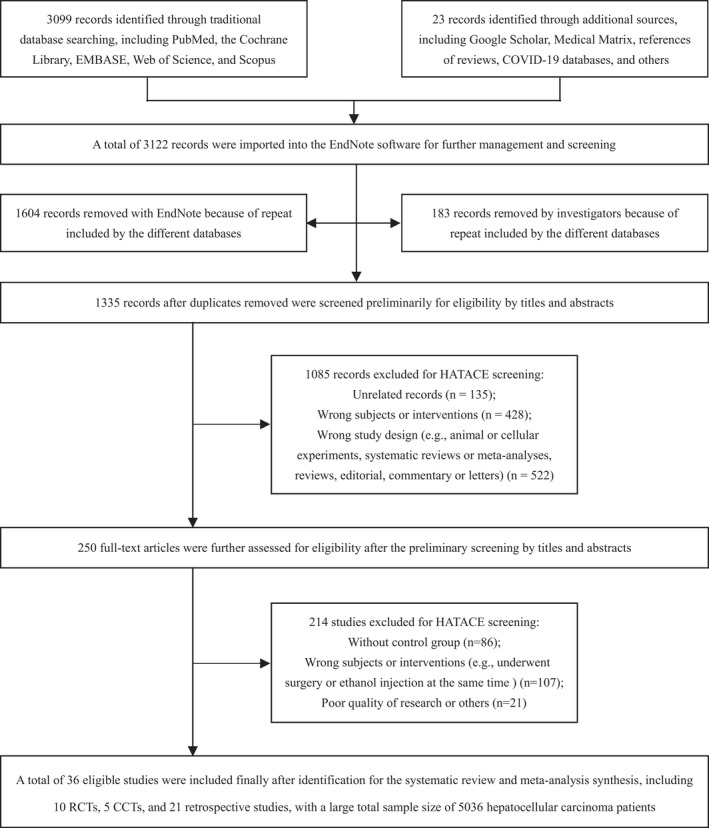
Identification flow chart of the studies to evaluate HATACE for HCC. CCT, controlled clinical trial; HATACE, hyperthermia ablation combined with transarterial chemoembolization; HCC, hepatocellular carcinoma; RCT, randomized controlled trial

Identification flow of the studies is exhibited as Figure 1 and Data S1. A total of 36 eligible studies^39^, ^40^, ^41^, ^42^, ^43^, ^44^, ^45^, ^46^, ^47^, ^48^, ^49^, ^50^, ^51^, ^52^, ^53^, ^54^, ^55^, ^56^, ^57^, ^58^, ^59^, ^60^, ^61^, ^62^, ^63^, ^64^, ^65^, ^66^, ^67^, ^68^, ^69^, ^70^, ^71^, ^72^, ^73^, ^74^ involving a large sample amount of 5036 patients were included finally for the systematic review and meta‐analysis (Figure 1, Table 1, Data S1). All of the included studies^39^, ^40^, ^41^, ^42^, ^43^, ^44^, ^45^, ^46^, ^47^, ^48^, ^49^, ^50^, ^51^, ^52^, ^53^, ^54^, ^55^, ^56^, ^57^, ^58^, ^59^, ^60^, ^61^, ^62^, ^63^, ^64^, ^65^, ^66^, ^67^, ^68^, ^69^, ^70^, ^71^, ^72^, ^73^, ^74^ were published in SCI journals and included in the Web of Science with good quality of reports. The main features of the included trials^39^, ^40^, ^41^, ^42^, ^43^, ^44^, ^45^, ^46^, ^47^, ^48^, ^49^, ^50^, ^51^, ^52^, ^53^, ^54^, ^55^, ^56^, ^57^, ^58^, ^59^, ^60^, ^61^, ^62^, ^63^, ^64^, ^65^, ^66^, ^67^, ^68^, ^69^, ^70^, ^71^, ^72^, ^73^, ^74^ are detailedly presented in Table 1 and Data S1.

**TABLE 1:** Assessment of the basic characteristics of the 36 included studies

Study (year)	Treatment	Nation	Study design	Research year range	Cases (*n*)	Age (years)	M/F (*n*)	Child–Pugh A/B/C (*n*)	Diameter (cm)
Chai NX 2021^39^	THA versus HA	America	CS	2010–2018	Total: 85; THA:21, HA:64	THA: 64.2 ± 7.2^c^; HA: 61.7 ± 8.9^c^	THA: 14/7; HA: 49/15	THA: 16/5/0; HA: 49/15/0	THA: 2.7 ± 1.0^c^, 21^d^(<3)^b^; HA: 2.2 ± 1.1^c^, 64^d^(<3)^b^
Zaitoun MMA 2021^40^	TM versus T versus M	Egypt	RCT	2017.1–2020.5	Total: 265; TM:89, T:84, M:92	TM: 52.1 ± 9.5^c^(48–76)^b^; T: 51.3 ± 9.2^c^(41–75)^b^; M: 53.8 ± 10.3^c^(38–72)^b^	TM:52/37; T:52/32; M:50/42	TM:80/9/0; T:71/13/0; M:78/14/0	TM: 3.7 ± 0.8^c^, 89^d^(3–5)^b^; T: 3.6 ± 0.8^c^, 84^d^(3.2–4.8)^b^; M: 3.9 ± 0.9^c^, 92^d^(3–5)^b^
Liu Y 2020^41^	TR versus T	China	PSMS	2008.10–2016.5	Total: 278; TR: 139, T: 139	TR: 56^a^ (28–78)^b^; T: 54^a^ (25–78)^b^	TR: 117/22; T: 112/27	TR: 89(A)/50(B or C); T: 89(A)/50(B or C)	TR: 63^d^(<5)^b^, 76^d^(≥5)^b^; T: 51^d^(<5)^b^, 88^d^(≥5)^b^
Li ZN 2020^42^	TM versus T	China	CS	2015.6–2017.5	Total: 51; TM: 23, T: 28	TM: 56^a^ (36–69)^b^; T: 52^a^ (34–65)^b^	TM: 15/8; T: 19/9	TM: 14/9/0; T: 18/10/0	TM: 11^d^(<3)^b^, 12^d^(3≥, <5)^b^; T: 9^d^(<3)^b^, 19^d^(3≥, <5)^b^
Chu HH 2019^43^	TR versus T versus R	Korea	PSMS	2000.3–2016.12	Total: 538; TR:109, T:314, R:115	TR: 58.4 ± 10.2^c^; T: 60.5 ± 10.6^c^; R: 61.1 ± 10.8^c^	TR:83/26; T:224/90; R:90/25	TR:93/16/0; T:83/32/0; R:254/60/0	TR: 3.8 ± 0.5^c^; T: 3.8 ± 0.5^c^; R: 3.5 ± 0.4^c^
Iezzi R 2019^44^	TR versus T	Italy	CCT	2010.1–2017.6	Total: 37; TR:21, T:16	TR: 65.7 ± 5.6^c^ (51–74)^b^; T: 63.1 ± 6.2^c^ (48–78)^b^	TR:15/6; T:12/4	TR:13/8/0; T:10/6/0	TR: 2^d^(2–3)^b^, 11^d^(3–5)^b^, 8^d^(>5)^b^; T: 2^d^(2–3)^b^, 9^d^(3–5)^b^, 5^d^(>5)^b^
Liu FR 2019^45^	TR versus T	China	CS	2005.1–2012.12	Total: 404; TR:209, T:195	TR: 59.2 ± 4.0^c^(18–75)^b^; T: 58.7 ± 4.0^c^(20–75)^b^	TR: 184/25; T: 165/30	TR: 189/20/0; T: 180/15/0	TR: 125^d^(≤3)^b^, 84^d^(>3)^b^; T: 114^d^(≤3)^b^, 81^d^(>3)^b^
Hirooka M 2018^46^	TR versus T	Japan	CS, MS	2000.1–2015.12	Total: 64; TR:32, T:32	TR: 69.5 ± 8.9^c^; T: 68.6 ± 8.9^c^	TR:25/7; T:28/4	TR:29/3/0; T:31/1/0	TR: 4.5 ± 2.4^c^; T: 4.3 ± 2.6^c^
Smolock AR 2018^47^	TM versus T	America	CS	2007–2016	Total: 47; TM:23, T:24	TM: 61^a^ (44–85)^b^; T: 64^a^ (43–76)^b^	TM:18/4; T:13/3	TM:14/9/0; T:14/7/3	TM: 4.2^a^ (3–5)^b^; T: 3.75^a^ (3–5)^b^
Wei YY 2018^48^	TM versus T	China	CS	2010.9–2015.8	Total: 81; TM:12, T:69	TM: 55 ± 11^c^; T: 51 ± 11^c^	TM:11/1; T:59/10	TM:5/7/0; T:15/47/7	Length, TM: 13.33 ± 1.37^c^, T: 13.21 ± 3.07^c^; Width, TM: 9.9 ± 0.89 ^c^, T: 10.20 ± 1.65^c^
Zhang RS 2018^49^	TM versus T	China	CS	2007.3–2016.4	Total: 150; TM:50, T:100	TM: 22^d^(≤55)^b^,28^d^(>55)^b^; T: 42^d^(≤55)^b^,58^d^(>55)^b^	TM:43/7; T:91/9	TM:46/4/0; T:94/6/0	TM: 36^d^(≤5)^b^, 14^d^(>5)^b^; T: 73^d^(≤5)^b^, 27^d^(>5)^b^
Zheng L 2018^50^	TM versus T	China	CS	2011.7–2015.4	Total: 258; TM:92, T:166	TM: 53.3 ± 8.2^c^; T: 54.6 ± 10.5^c^	TM:79/13; T:143/23	Unclear	TM:9.1 ± 2.8^c^, 48^d^(≤10)^b^, 44^d^(>10)^b^; T: 8.5 ± 2.5^c^, 94^d^(≤10)^b^, 72^d^(>10)^b^
Chen QF 2017^51^	TM versus T	China	PSMS	2014.6–2015.12	Total: 144; TM:48, T:96	TM: 58.8 ± 9.6^c^, 24^d^(≥60)^b^, 24^d^(<60)^b^; T: 59.7 ± 10.5^c^, 49^d^(≥60)^b^, 47^d^(<60)^b^	TM:28/20; T:54/42	TM:39/9/0; T:84/12/0	TM: 27.4 ± 10.9^c^; T: 28.8 ± 12.5^c^
Jiang FQ 2017^52^	TR versus T	China	RCT	2012.6–2014.6	Total: 106; TR:53, T:53	TR: 63 ± 7^c^; T: 63 ± 6^c^	TR:30/23; T:31/22	TR:29/21/3; T:28/20/5	Unclear
Hyun D 2016^53^	TR versus T	Korea	CS	2007.1–2010.12	Total: 91; TR:37, T:54	TR: 57.7 ± 7.7^c^; T: 59.5 ± 9.5^c^	TR: 31/6; T: 42/12	TR: 34/3/0; T: 45/9/0	TR: 28^d^(≤2)^b^, 9^d^(>2–3)^b^; T: 32^d^(≤2)^b^, 22^d^(>2–3)^b^
Li W 2016^54^	TM versus T	China	CS	2005.12–2015.12	Total: 84; TM:42, T:42	TM: 48; T: 50	Unclear	Unclear	Unclear
Sheta E 2016^55^	THA versus T	Egypt	RCT	Unclear	Total: 50; TM:20, TR:20, T:10	Unclear	Unclear	TM:8/2/0; TR:16/4/0; T:14/6/0	TM: 5.15 ± 0.27^c^(4.8–5.6)^b^; TR: 4.87 ± 0.42^c^(4.2–5.6)^b^; T: 4.82 ± 0.57^c^(4–6)^b^
Song MJ 2016^56^	TR versus T versus R	Korea	CS	2004.12–2010.2	Total: 201; TR:87, T:71, R:43	TR: 60.4^a^(29.1–78.0)^b^; T: 60.0^a^(23.0–87.2)^b^; R: 62.0^a^(35.0–88.0)^b^	TR:70/17; T:53/18; R:31/12	TR:80/7/0; T:68/3/0; R:37/6/0	TR: 2.5^a^(1.0–4.6)^b^, 64^d^(<3)^b^, 23^d^(≥3)^b^; T: 2.5^a^(1.0–4.7)^b^, 44^d^(<3)^b^, 27^d^(≥3)^b^; R: 2.2^a^(1.3–4.7)^b^, 33^d^(<3)^b^, 10^d^(≥3)^b^
Tang CW 2016^57^	TR versus T versus R	China	CS	2009.6–2012.6	Total: 132; TR:40, T:43, R:49	TR: 48.28 ± 13.48^c^; T: 45.84 ± 15.08^c^; R: 47.14 ± 13.27^c^	TR:29/11; T:33/10; R:34/15	TR:18/22/0; T:19/24/0; R:22/27/0	TR: 5.35 ± 1.10^c^; T: 5.64 ± 1.41^c^; R: 5.78 ± 1.35^c^
Liu HC 2014^58^	TR versus T	China	CCT	2005.6–2011.6	Total: 88, TR:45, T:43	TR: 45–75^b^; T: 44–78^b^	TR:36/9; T:34/9	TR:13/20/12; T:10/23/10	TR: 4–15^b^; T: 5–14^b^
Yin X 2014^59^	TR versus T	China	CS	2005.1–2011.12	Total: 211; TR:55, T:156	TR: 19^d^(≤50)^b^,36^d^(>50)^b^; T: 54^d^(≤50)^b^,102^d^(>50)^b^	TR:47/8; T:138/18	TR:48/7/0; T:136/20/0	TR: 5.9^a^ (5–8)^b^; T: 6.0^a^ (5–8)^b^
Yi YX 2014^60^	THA versus HA	China	RCT	2008.6–2010.6	Total: 94; THA:47, HA:47	THA: 56.8 ± 5.6^c^; HA: 55.9 ± 5.4^c^	THA: 37/10; HA: 34/13	THA: 45/2/0; HA: 44/3/0	THA: 3.45 ± 1.45^c^, 22^d^(≤3)^b^, 25^d^(>3)^b^; HA: 3.38 ± 1.33^c^, 20^d^(≤3)^b^, 27^d^(>3)^b^
Peng ZW 2013^61^	TR versus R	China	RCT	2006.10–2009.6	Total: 189; TR:94, R:95	TR: 53.3 ± 11.0^c^; R: 55.3 ± 13.3^c^	TR: 75/19; R:71/24	TR: 90/4/0; R: 90/5/0	TR: 3.47 ± 1.44^c^, 43^d^(≤3)^b^, 51^d^(>3)^b^; R: 3.39 ± 1.35^c^, 46^d^(≤3)^b^, 49^d^(>3)^b^
Xu LF 2013^62^	TM versus T	China	CS	2004.1–2011.12	Total: 136; TM:56, T:80	TM: 54.50 ± 12.95^c^; T: 53.10 ± 14.80^c^	TM:48/8; T: 73/7	Unclear	TM: 9.48 ± 2.36^c^; T: 10.16 ± 2.09^c^
Kim JW 2012^63^	TR versus R	Korea	CS	2001.6–2008.9	Total: 314; TR:83, R:231	TR: 59.7 ± 10.4^c^; R: 58.0 ± 10.1^c^	TR: 69/14; R:182/49	TR: 67/16/0; R: 170/61/0	TR: 2.5 ± 0.3^c^; R: 2.4 ± 0.3^c^
Peng ZW 2012^64^	TR versus R	China	RCT	2002.1–2006.12	Total: 139; TR:69, R:70	TR: 57.5 ± 10.0^c^ (19–75)^b^; R: 55.1 ± 9.5^c^ (22–75)^b^	TR: 59/9; R: 55/15	TR: 60/9/0; R: 59/11/0	TM: 41^d^(≤3)^b^, 28^d^(3.1–5.0)^b^; T: 46^d^(≤3)^b^, 24^d^(3.1–5.0)^b^
Kim JH 2011^65^	TR versus R	Korea	CS	2000.3–2010.4	Total: 123; TR:57, R:66	TR: 57.9 ± 10.5^c^; R: 58.7 ± 10.7^c^	TR: 45/12; R: 51/15	TR: 49/8/0; R: 43/23/0	TR: 3.8 ± 0.5^c^; R: 3.7 ± 0.5^c^
Liu C 2011^66^	TM versus T	China	CCT	2004.5–2006.12	Total: 34; TM:16, T:18	TM: 52.1 ± 14.5^c^; T: 51.9 ± 13.6^c^	TM:14/2; T:15/3	TM:8/7/1; T:9/8/1	TM: 6.8 ± 1.5^a^; T: 6.7 ± 1.5^a^
Morimoto M 2010^67^	TR versus R	Japan	RCT	2005.8–2009.4	Total: 37; TR:19, R:18	TR: 70^c^ (57–78)^b^; R: 73^c^ (48–84)^b^	TR: 15/4; R: 12/6	TR: 18/1/0; R: 16/2/0	TR: 3.6 ± 0.7^c^; R: 3.7 ± 0.6^c^
Shibata T 2009^68^	TR versus R	Japan	RCT	2003.7–2007.12	Total: 89; TR:46, R:43	TR: 67.2 ± 8.9^c^ (45–83)^b^; R: 69.8 ± 8.0^c^ (44–87)^b^	TR: 31/15; R: 33/10	TR: 32/14/0; R: 33/10/0	TR: 1.7 ± 0.6^c^ (0.9–3.0)^b^, 8^d^(<1.0)^b^, 26^d^(1.0–2.0)^b^, 15^d^(>2.0)^b^; R: 1.6 ± 0.5^c^ (0.8–2.6)^b^, 5^d^(<1.0)^b^, 31^d^(1.0–2.0)^b^, 8^d^(>2.0)^b^
Yang W 2009^69^	TR versus T versus R	China	CS	2000.7–2007.1	Total: 103; TR:31, T:35, R:37	TR: 57.8^c^(43–78)^b^; T: 51.2^c^(30–74)^b^; R: 58.3^c^(38–80)^b^	TR:24/7; T:30/5; R:27/10	TR:20/10/1; T:21/13/1; R:23/13/1	TR: 3.5^c^(1.7–7.3)^b^; T: 3.6^c^(1.2–8.0)^b^; R: 3.8^c^(2–6.4)^b^
Yamagiwa K 2008^70^	TR versus T	Japan	CS	1995.1–2004.12	Total: 201; TR:115, T:86	Unclear	Unclear	Unclear	Unclear
Yang P 2008^71^	TR versus T versus R	China	RCT	2004.2–2006.7	Total: 47; TR:24, T:11, R:12	TR: 59.1 ± 11.4^c^; T: 57.6 ± 11.8^c^; R: 61.0 ± 10.4^c^	TR:18/6; T:8/3; R:8/4	TR:11/5/1; T:10/5/0; R:8/6/1	TR: 6.6 ± 0.6^c^; T: 6.4 ± 1.0^c^; R: 5.2 ± 0.4^c^
Wang YB 2007^72^	TR versus T	China	CCT	2003.10–2004.12	Total: 87; TR:43, T:40	TR: 12^d^(≤50)^b^,11^d^(51–60)^b^,13^d^(61–70)^b^,7^d^(>70)^b^; T: 7^d^(≤50)^b^,18^d^(51–60)^b^,10^d^(61–70)^b^,5^d^(>70)^b^	TR:32/11; T:34/6	TR:34/9/0; T:32/8/0	TR: 20^d^(≤3.0)^b^, 7^d^(3.1–3.5)^b^, 16^d^(>3.5)^b^; T: 18^d^(≤3.0)^b^, 9^d^(3.1–3.5)^b^, 23^d^(>3.5)^b^
Aikata H 2006^73^	TR versus R	Japan	RCT	Unclear	Total: 44;TR:21, R:23	Unclear	Unclear	Unclear	Unclear
Shen SQ 2005^74^	TR versus R	China	CCT	2001.9–2004.6	Total: 34;TR:18, R:16	TR: 52.7^c^ (20–72)^b^; R: 56.1^c^(36–75)^b^	TR: 5/13; R: 3/13	TR: 4/14/0; R: 6/10/0	TR: 5.6^a^ (2.2–15.8)^b^13^d^(≤5)^b^, 5^d^(>5)^b^; R: 5.0^a^ (2.3–12.3)^b^13^d^(≤5)^b^, 3^d^(>5)^b^

Abbreviations: CCT, controlled clinical trial, prospective; CS, case–control study, or retrospective cohort study; F, female; HA: hyperthermia ablation, RFA or MWA; M, male; M: microwave ablation, MWA; MS, multicenter study; PSMS, propensity score matching study; R: radiofrequency ablation, RFA; RCT, randomized controlled trial; T: transarterial chemoembolization, TACE; THA: HA combined with TACE, HATACE; TM: TACE combined with MWA; TR: TACE combined with RFA.

^a^ Median.

^b^ Range.

^c^ Average.

^d^ Number of people.

**FIGURE 2:**
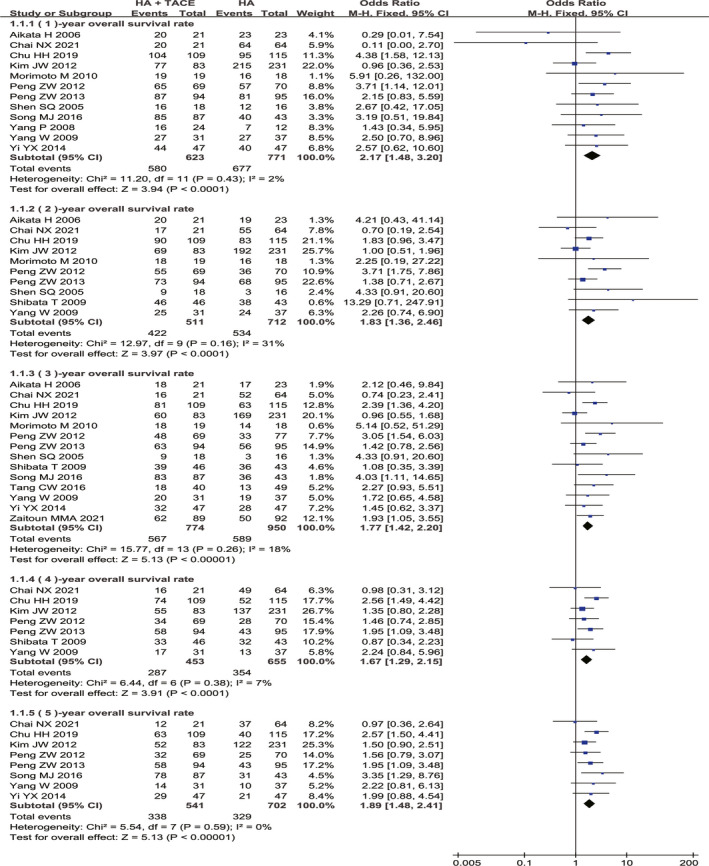
Meta‐analysis of OS in HATACE group compared with HA alone. CI, confidence interval; HA, hyperthermia ablation; HATACE, HA combined with TACE; M–H, Mantel–Haenszel; OS, overall survival; TACE, transarterial chemoembolization

The meta‐analyses results showed that compared with HA alone, HATACE was associated with a significant improvement in the OS rate at 1 year (OR = 2.17, 95% CI = 1.48–3.20, *p *< 0.0001), 2 years (OR = 1.83, 95% CI = 1.36–2.46, *p *< 0.0001), 3 years (OR = 1.77, 95% CI = 1.42–2.20, *p *< 0.00001), 4 years (OR = 1.67, 95% CI = 1.29–2.15, *p *< 0.0001), and 5 years (OR = 1.89, 95% CI = 1.48–2.41, *p *< 0.00001; Figure 2). No significant differences were found between the HATACE group and HA alone group with respect to the incidences of severe liver damage (OR = 1.36, 95% CI = 0.46–4.03, *p* = 0.58), ascites (OR = 1.31, 95% CI = 0.48–3.60, *p* = 0.60), abdominal infection (OR = 1.01, 95% CI = 0.20–5.05, *p* = 0.99), abdominal pain (OR = 1.09, 95% CI = 0.78–1.53, *p* = 0.62), bleeding (OR = 1.38, 95% CI = 0.64–2.98, *p* = 0.41), pleural effusion (OR = 0.97, 95% CI = 0.33–2.84, *p* = 0.96), fever (OR = 1.23, 95% CI = 0.84–1.82, *p* = 0.29), and nausea and vomiting (OR = 1.97, 95% CI = 0.77–5.08, *p* = 0.16; Table 2).

**TABLE 2:** Meta‐analysis of HATACE group compared with monotherapy group

Outcome	Included studies	HATACE		Monotherapy		Heterogeneity		Statistical method	Results of meta‐an alysis	
		*n*	*N*	*n*	*N*	I^2^	*p*		OR(95%CI)	*p*
HATACE versus HA										
Severe liver damage	7^40^, ^43^, ^63^, ^64^, ^65^, ^68^, ^74^	4	471	3	633	0%	0.89	OR (M‐H, Fixed, 95%CI)	1.36 (0.46, 4.03)	0.58
Ascites	3^60^, ^61^, ^64^	9	210	7	212	0%	0.97	OR (M‐H, Fixed, 95%CI)	1.31 (0.48, 3.60)	0.60
Abdominal infection	3^60^, ^61^, ^64^	2	210	2	212	0%	0.63	OR (M‐H, Fixed, 95%CI)	1.01 (0.20, 5.05)	0.99
Abdominal pain	5^40^, ^60^, ^61^, ^64^, ^67^	151	318	147	322	25%	0.26	OR (M‐H, Fixed, 95%CI)	1.09 (0.78, 1.53)	0.62
Bleeding	10^39^, ^43^, ^57^, ^60^, ^61^, ^63^, ^64^, ^65^, ^68^, ^69^	11	597	11	817	0%	0.89	OR (M‐H, Fixed, 95%CI)	1.38 (0.64, 2.98)	0.41
Pleural effusion	4^60^, ^61^, ^67^, ^74^	7	178	7	176	0%	0.89	OR (M‐H, Fixed, 95%CI)	0.97 (0.33, 2.84)	0.96
Fever	4^40^, ^60^, ^61^, ^64^	79	299	69	304	13%	0.33	OR (M‐H, Fixed, 95%CI)	1.23 (0.84, 1.82)	0.29
Nausea and vomiting	4^40^, ^60^, ^61^, ^64^	85	299	53	304	73%	0.01	OR (M‐H, Random, 95%CI)	1.97 (0.77, 5.08)	0.16
HATACE versus TACE										
1‐year OS rate	18^41^, ^43^, ^45^, ^46^, ^48^, ^49^, ^50^, ^51^, ^53^, ^54^, ^56^, ^58^, ^59^, ^62^, ^66^, ^71^	943	1060	1178	1568	46%	0.02	OR (M‐H, Fixed, 95%CI)	2.93 (2.29, 3.74)	<0.00001
2‐year OS rate	9^43^, ^50^, ^51^, ^53^, ^54^, ^58^, ^66^, ^69^, ^70^	400	535	530	854	59%	0.01	OR (M‐H, Random, 95%CI)	2.83 (1.72, 4.66)	<0.0001
3‐year OS rate	14^40^, ^43^, ^45^, ^46^, ^49^, ^50^, ^53^, ^56^, ^57^, ^58^, ^59^, ^62^, ^69^, ^70^	677	1047	626	1459	68%	0.0001	OR (M‐H, Random, 95%CI)	3.16 (2.22, 4.50)	<0.00001
4‐year OS rate	4^43^, ^58^, ^69^, ^70^	198	300	223	478	88%	<0.00001	OR (M‐H, Random, 95%CI)	3.50 (1.19, 10.25)	0.02
5‐year OS rate	9^43^, ^45^, ^46^, ^49^, ^56^, ^59^, ^62^, ^69^, ^70^	371	744	316	1069	82%	<0.00001	OR (M‐H, Random, 95%CI)	3.54 (1.96, 6.37)	<0.0001
Severe liver damage	8^40^, ^42^, ^46^, ^49^, ^53^, ^59^, ^69^, ^70^	5	489	20	684	0%	0.93	OR (M‐H, Fixed, 95%CI)	0.41 (0.18, 0.98)	0.04
Ascites	5^45^, ^53^, ^58^, ^70^, ^72^	20	451	32	420	0%	0.63	OR (M‐H, Fixed, 95%CI)	0.54 (0.30, 0.98)	0.04
Abdominal infection	4^42^, ^43^, ^45^, ^50^	10	433	7	703	0%	0.81	OR (M‐H, Fixed, 95%CI)	2.13 (0.77, 5.84)	0.14
Abdominal pain	5^40^, ^43^, ^45^, ^49^, ^50^	237	549	304	859	45%	0.12	OR (M‐H, Fixed, 95%CI)	1.07 (0.82, 1.40)	0.62
Bleeding	10^43^, ^45^, ^46^, ^49^, ^50^, ^57^, ^58^, ^59^, ^70^, ^72^	15	849	20	1286	0%	0.50	OR (M‐H, Fixed, 95%CI)	0.93 (0.49, 1.78)	0.83
Pleural effusion	5^42^, ^45^, ^50^, ^59^, ^72^	23	422	26	585	20%	0.29	OR (M‐H, Fixed, 95%CI)	1.06 (0.60, 1.85)	0.85
Fever	4^40^, ^45^, ^49^, ^50^	173	440	247	545	61%	0.05	OR (M‐H, Random, 95%CI)	0.88 (0.52, 1.50)	0.64
Nausea and vomiting	4^40^, ^45^, ^49^, ^50^	149	440	196	545	40%	0.17	OR (M‐H, Fixed, 95%CI)	0.96 (0.72, 1.27)	0.77

Abbreviations: CI, confidence interval; HA, hyperthermia ablation; M–H, Mantel–Haenszel; OS, overall survival; TACE, transarterial chemoembolization.

The results demonstrated that the OS rates were significantly higher with HATACE than TACE alone at 1, 2, 3, 4, and 5 years. Compared with TACE alone, HATACE was associated with significant reduction in the incidences of severe liver damage (OR = 0.41, 95% CI = 0.18–0.98, *p* = 0.04) and ascites (OR = 0.54, 95% CI = 0.30–0.98, *p* = 0.04). No significant differences were observed between HATACE group and TACE alone group with respect to the incidences of abdominal infection, abdominal pain, bleeding, pleural effusion, fever, and nausea and vomiting (Table 2).

### 3.2 Results of the sensitivity analysis with RCTs

**FIGURE 3:**
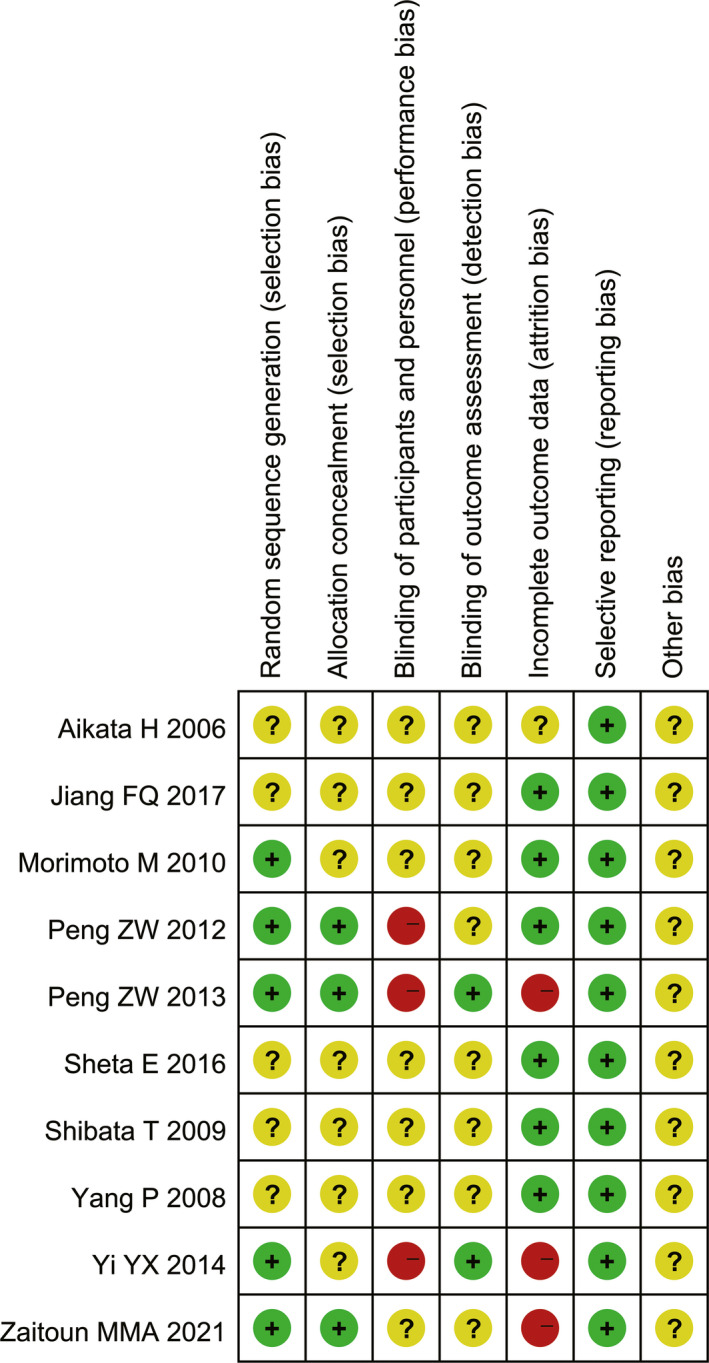
Risk of bias summary before correction: each risk of bias item for each RCT of sensitivity analysis. Green: low risk of bias; Yellow: unclear risk of bias; Red: high risk of bias

**FIGURE 4:**
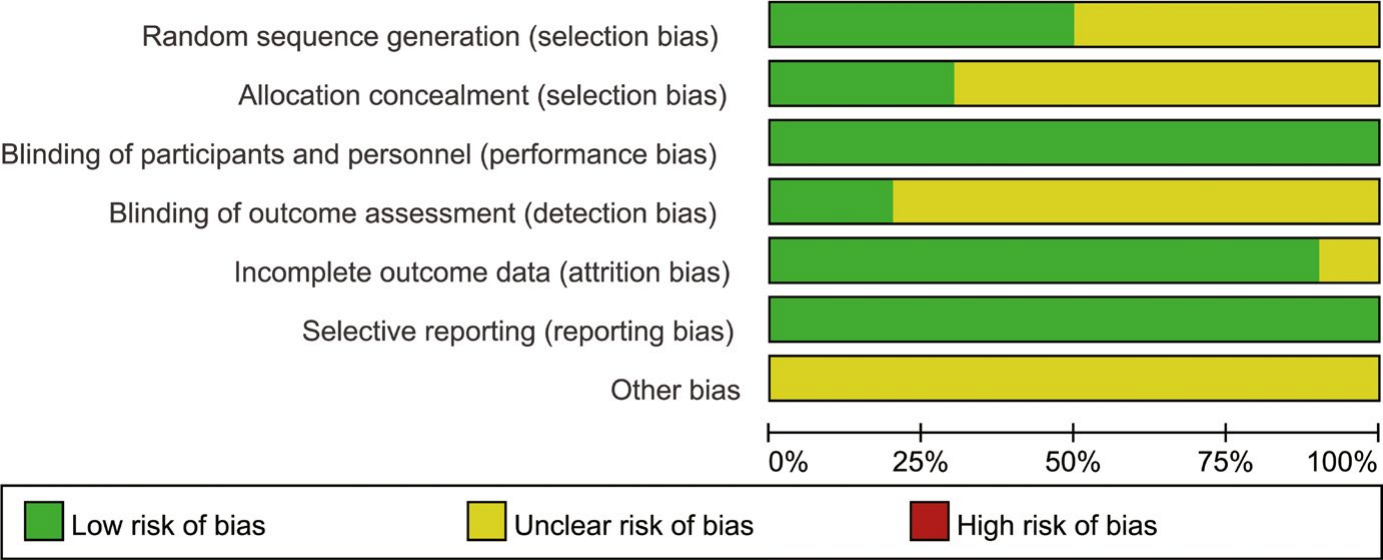
Risk of bias item presented after correction as percentages across all RCTs of sensitivity analysis. Green: low risk of bias; Yellow: unclear risk of bias; Red: high risk of bias

The sensitivity analysis of RCTs was performed to test the robustness of our findings derived from the meta‐analyses above. A total of 10 RCTs^40^, ^52^, ^55^, ^60^, ^61^, ^64^, ^67^, ^68^, ^71^, ^73^ were identified from the 36 included studies,^39^, ^40^, ^41^, ^42^, ^43^, ^44^, ^45^, ^46^, ^47^, ^48^, ^49^, ^50^, ^51^, ^52^, ^53^, ^54^, ^55^, ^56^, ^57^, ^58^, ^59^, ^60^, ^61^, ^62^, ^63^, ^64^, ^65^, ^66^, ^67^, ^68^, ^69^, ^70^, ^71^, ^72^, ^73^, ^74^ which contained 6 studies^60^, ^61^, ^64^, ^67^, ^68^, ^73^ of HATACE versus HA monotherapy, 2 studies^52^, ^55^ of HATACE versus TACE monotherapy, and 2 studies^40^, ^71^ of HATACE versus HA (or TACE) monotherapy. Assessment list of methodological quality of all RCTs is summarized in Table 3. The risk of bias in each RCT was further assessed with figures by the RevMan software according to the Cochrane Collaboration's tool for randomized trials.^38^ Details of the analysis and correction for risk of bias assessment are presented in the Data S1. Based on the comprehensive analysis of risk of bias in RCTs,^40^, ^52^, ^55^, ^60^, ^61^, ^64^, ^67^, ^68^, ^71^, ^73^ we had a high degree of confidence in getting reliable results from the sensitivity analysis (Figure 3 and Figure 4).

**TABLE 3:** Assessment of the methodological quality of the RCTs for sensitivity analysis

Study and year	Study design	Randomization	Allocated concealment	Baseline control	Blinding		Incomplete outcome data	Selective outcome reporting	Other biases
					Participants and personnel	Outcome assessment			
Zaitoun MMA 2021^40^	RCT	Serially numbered containers	Serially numbered containers	Adequate	Unclear	Unclear	YES (*N*=13)	NO	Unclear
Jiang FQ 2017^52^	RCT	Unclear	Unclear	Adequate	Unclear	Unclear	NO	NO	Unclear
Sheta E 2016^55^	RCT	Unclear	Unclear	Adequate	Unclear	Unclear	NO	NO	Unclear
Yi YX 2014^60^	RCT	Computer‐generated	Unclear	Adequate	NO	YES	YES (*N*=1), ITT	NO	Unclear
Peng ZW 2013^61^	RCT	Computer‐generated	Central	Adequate	NO	YES	YES (*N*=3), ITT	NO	Unclear
Peng ZW 2012^64^	RCT	Computer‐generated	Envelopes	Adequate	NO	Unclear	NO	NO	Unclear
Morimoto M 2010^67^	RCT	Computer‐generated	Unclear	Adequate	Unclear	Unclear	NO	NO	Unclear
Shibata T 2009^68^	RCT	Unclear	Unclear	Adequate	Unclear	Unclear	NO	NO	Unclear
Yang P 2008^71^	RCT	Unclear	Unclear	Adequate	Unclear	Unclear	NO	NO	Unclear
Aikata H 2006^73^	RCT	Unclear	Unclear	Adequate	Unclear	Unclear	Unclear	NO	Unclear

Abbreviations: *N*, Number of loss of follow‐up: ITT, intention‐to‐treat analysis; RCT, randomized controlled trial.

**FIGURE 5:**
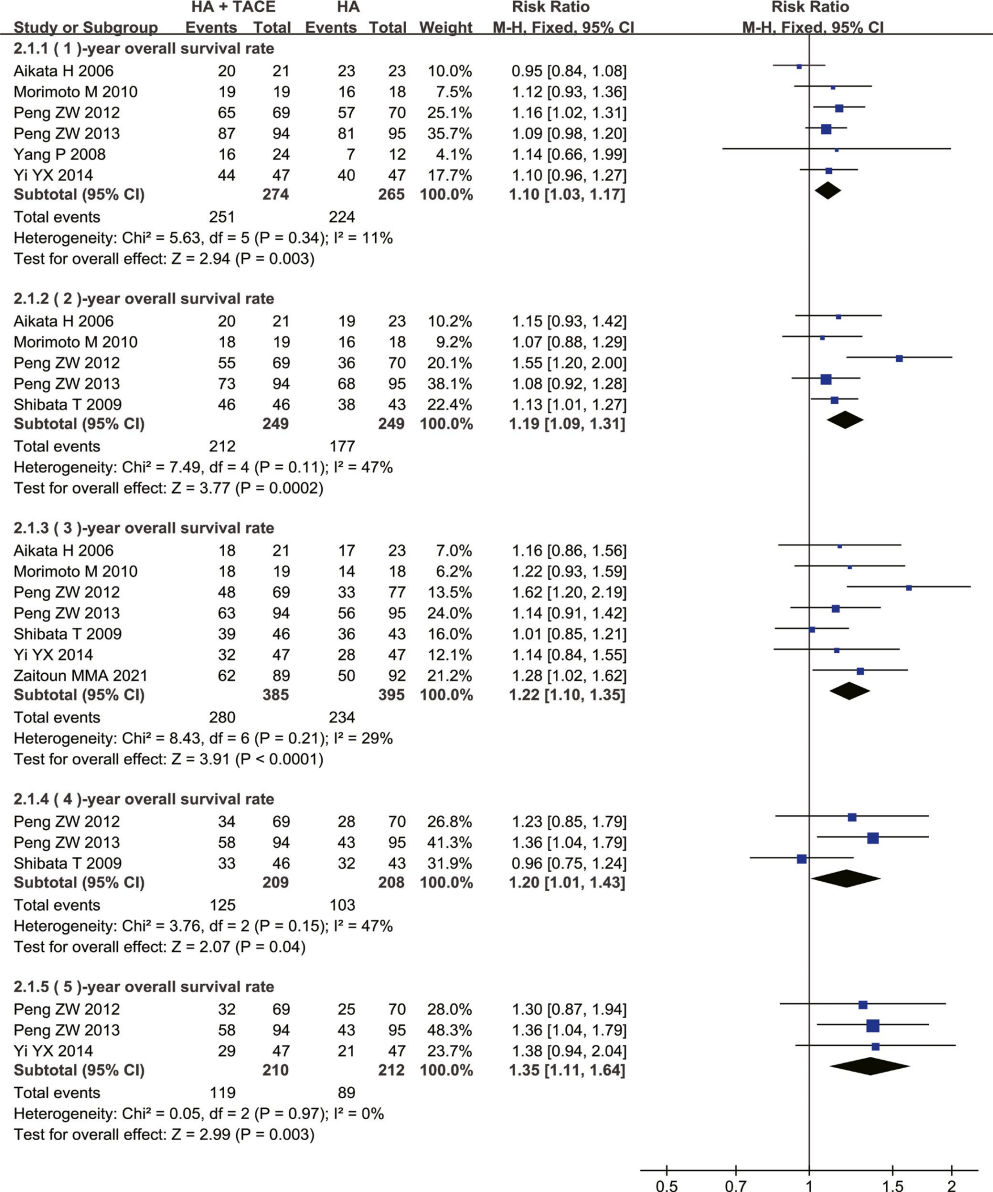
Sensitivity analysis of OS for HATACE group compared with HA group. CI, confidence interval; HA, hyperthermia ablation; HATACE, HA combined with TACE; M–H, Mantel–Haenszel; OS, overall survival; TACE, transarterial chemoembolization

The sensitivity analysis for HATACE versus HA was completed with a total of eight RCTs.^40^, ^60^, ^61^, ^64^, ^67^, ^68^, ^71^, ^73^ The meta‐analyses results demonstrated that HATACE significantly improved the OS rate over HA alone at 1 year (RR = 1.10, 95% CI = 1.03–1.17, *p* = 0.003), 2 years (RR = 1.19, 95% CI = 1.09–1.31, *p* = 0.0002), 3 years (RR = 1.22, 95% CI = 1.10–1.35, *p *< 0.0001), 4 years (RR = 1.20, 95% CI = 1.01–1.43, *p* = 0.04), and 5 years (RR = 1.35, 95% CI = 1.11–1.64, *p* = 0.003; Figure 5). There were no significant differences between the HATACE group and HA alone group with respect to the incidences of severe liver damage (RR = 2.98, 95% CI = 0.48–18.71, *p* = 0.24), ascites (RR = 1.30, 95% CI = 0.49–3.40, *p* = 0.60), abdominal infection (RR = 1.01, 95% CI = 0.21–4.95, *p* = 0.99), abdominal pain (RR = 1.04, 95% CI = 0.89–1.21, *p* = 0.62), bleeding (RR = 1.49, 95% CI = 0.43–5.19, *p* = 0.53), pleural effusion (RR = 0.99, 95% CI = 0.33–2.99, *p* = 0.99), fever (RR = 1.16, 95% CI = 0.88–1.52, *p* = 0.29), and nausea and vomiting (RR = 1.59, 95% CI = 0.80–3.17, *p* = 0.19; Table 4). The findings of HATACE versus HA were identified as reliable and stable on the basis of the results of sensitivity meta‐analyses with RCTs (Figure 5 and Table 4).

**TABLE 4:** Sensitivity analysis of the safety for HATACE group compared with HA group

Outcome	Included studies	HATACE		HA		Heterogeneity		Statistical method	Results of meta‐analysis	
		*n*	*N*	*n*	*N*	I^2^	*p*		RR (95%CI)	*p*
Severe liver damage	3^40^, ^64^, ^68^	3	204	0	205	0%	1.00	RR (M‐H, Fixed, 95%CI)	2.98 (0.48, 18.71)	0.24
Ascites	3^60^, ^61^, ^64^	9	210	7	212	0%	0.97	RR (M‐H, Fixed, 95%CI)	1.30 (0.49, 3.40)	0.60
Abdominal infection	3^60^, ^61^, ^64^	2	210	2	212	0%	0.64	RR (M‐H, Fixed, 95%CI)	1.01 (0.21, 4.95)	0.99
Abdominal pain	5^40^, ^60^, ^61^, ^64^, ^67^	151	318	147	322	12%	0.34	RR (M‐H, Fixed, 95%CI)	1.04 (0.89, 1.21)	0.62
Bleeding	4^60^, ^61^, ^64^, ^68^	5	256	3	255	0%	0.66	RR (M‐H, Fixed, 95%CI)	1.49 (0.43, 5.19)	0.53
Pleural effusion	3^60^, ^61^, ^67^	6	160	6	160	0%	0.74	RR (M‐H, Fixed, 95%CI)	0.99 (0.33, 2.99)	0.99
Fever	4^40^, ^60^, ^61^, ^64^	79	299	69	304	3%	0.38	RR (M‐H, Fixed, 95%CI)	1.16 (0.88, 1.52)	0.29
Nausea and vomiting	4^40^, ^60^, ^61^, ^64^	85	299	53	304	72%	0.01	RR (M‐H, Random, 95%CI)	1.59 (0.80, 3.17)	0.19

Abbreviations: CI, confidence interval; HA, hyperthermia ablation; M–H, Mantel–Haenszel; TACE, transarterial chemoembolization.

The sensitivity analysis for HATACE versus TACE was completed with a total of four RCTs.^40^, ^52^, ^55^, ^71^ We could not finish the sensitivity meta‐analysis of RCTs because of the limited studies and insufficient data, so qualitative synthetic evaluation was carried out for the sensitivity analysis of HATACE versus TACE. The synthetic results of the four RCTs^40^, ^52^, ^55^, ^71^ revealed that compared with TACE alone, HATACE was associated with significant improvement in the efficacy and significant reduction in the incidences of adverse reaction and complication, which is in conformity with the meta‐analyses results of HATACE versus TACE above.

### 3.3 Results of the subgroup analysis for small HCC

The subgroup analyses results for small HCC demonstrated that there were no significant differences between the HATACE group and HA alone group with respect to the OS rates at 1, 2, 3, 4, and 5 years (*p* ≥ 0.05; Table 5).

**TABLE 5:** Subgroup analysis results of HATACE compared with HA alone for small HCC

Outcome	Included studies	HATACE		HA		Heterogeneity		Statistical method	Results of meta‐analysis	
		*n*	*N*	*n*	*N*	I^2^	*p*		OR(95%CI)	*p*
1‐year OS rate	6^39^, ^56^, ^63^, ^64^, ^69^, ^73^	230	242	383	411	26%	0.24	OR (M‐H, Fixed, 95%CI)	1.34 (0.68, 2.63)	0.40
2‐year OS rate	4^39^, ^63^, ^68^, ^73^	152	171	304	361	38%	0.18	OR (M‐H, Fixed, 95%CI)	1.27 (0.74, 2.20)	0.39
3‐year OS rate	7^39^, ^56^, ^63^, ^64^, ^68^, ^69^, ^73^	229	288	336	454	0%	0.62	OR (M‐H, Fixed, 95%CI)	1.21 (0.84, 1.76)	0.31
4‐year OS rate	3^39^, ^63^, ^68^	104	150	218	338	0%	0.69	OR (M‐H, Fixed, 95%CI)	1.18 (0.77, 1.81)	0.44
5‐year OS rate	5^39^, ^56^, ^63^, ^64^, ^69^	148	221	211	388	0%	0.43	OR (M‐H, Fixed, 95%CI)	1.44 (1.00, 2.07)	0.05

Abbreviations: CI, confidence interval; HA, hyperthermia ablation; M–H, Mantel–Haenszel; OS, overall survival; TACE, transarterial chemoembolization.

## 4 DISCUSSION

The statistics showed that liver cancer was the third leading cause of cancer death worldwide in 2020, with about 906,000 new cases and 830,000 deaths annually.^75^, ^76^ In addition, there were approximately half cases and deaths of the total number worldwide distributed in China.^75^, ^76^ There is some debate that compared with the monotherapy of HA (or TACE) for treating HCC, whether HATACE could improve the efficacy meanwhile without increasing (or even reducing) toxicity and complications. This question should be definitively answered by the comprehensive systematic review and meta‐analysis results.

The synergistic effects of combined HA and TACE may overcome their own limitations and improve the therapeutic outcomes.^35^ The results of meta‐analyses demonstrated that the oncologic outcomes of HATACE were markedly superior to those of HA or TACE alone: (i) Compared with HA monotherapy, HATACE could significantly improve the OS rates of 1, 2, 3, 4, and 5 years, what is more, without increasing the incidences of adverse effects and complications. The robustness of the results was tested by the meta‐analysis of RCTs, and the results of sensitivity meta‐analysis confirmed that all of the measurement outcomes are reliable evidence. Therefore, the results of HATACE versus HA manifested that adjuvant TACE is necessary and complementary in the HA‐based integrated therapy. (ii) Compared with TACE monotherapy, HATACE could significantly improve the OS rates without increasing the incidences of abdominal infection, abdominal pain, bleeding, pleural effusion, fever, and nausea and vomiting; more importantly, HATACE was associated with significant reduction in the incidences of severe liver damage and ascites. These findings are in conformity with the sensitivity analysis results of qualitative synthetic evaluation with the data of RCTs. Therefore, the results of HATACE versus TACE manifested that HA plays a significant synergistic role in HATACE; most important of all, HA is confirmed to be effective in reducing the toxicity of TACE and protecting liver function to some extent.

There are two sides as coins in the relation between TACE and HCC patients’ systemic function status (including immunity). (i) Although systemic chemotherapy leads to immunosuppression, minimally invasive TACE controls it to a minimum via its substantively limited dose and focally delivered administration^35^, ^77^; (ii) TACE is beneficial to improve patient's holistic status (including immunity) because cancerous damage to the body (including immunosuppression caused by cancer) is ameliorated after reducing the cancer quantity^77^, ^78^, ^79^, ^80^; and (iii) The synthetical risk–benefit result of TACE, depending on the balance of the two aspects above, is superior to majority of the therapies for HCC, especially when the synergistic advantages were unlocked with HA combination.^35^, ^81^ It is also in line with the meta‐analysis results that HATACE was associated with significant improvement in the OS rates and significant reduction in the incidences of severe liver damage and ascites.

HATACE has a broad clinical applications for different stages of HCC based on the studies for systematic review, including early or very early stage^39^, ^42^, ^44^, ^47^, ^53^, ^56^ (Barcelona Clinic Liver Cancer [BCLC] stage 0 & A), intermediate stage^42^, ^45^, ^46^, ^47^, ^48^, ^49^ (BCLC stage B), and advanced stage^41^, ^47^, ^48^, ^54^ (BCLC stage C & D). However, conflicting conclusions exist among some different studies^39^, ^56^, ^63^, ^64^, ^68^, ^69^, ^73^ in regard to the survival benefit from HATACE compared with HA monotherapy for small (≤3 cm) HCC. To explore the potential different benefits associated with the different size classification of HCC, subgroup analyses were carried out for small HCC to compare HATACE with HA alone. The results revealed that the survival benefit of additional TACE is very limited without statistical significance for the patients with small HCC. Therefore, HATACE is more effective and befitting for non‐small‐sized (>3 cm) HCC than HA monotherapy.

Verna et al.^82^ indicated that all non‐essential studies were halted when the COVID‐19 pandemic started, and COVID‐19 should become the preferred research subject during this unprecedented pandemic for rescuing patients in disaster. Mancilla‐Galindo et al.^26^ presented a novel idea of mild hyperthermia (thermotherapy) as a potential therapy for patients with mild‐to‐moderate COVID‐19 to prevent disease progression. Hyperthermia therapy (including HA) could improve the immunity of cancer patients,^23^, ^24^, ^25^, ^26^, ^27^, ^28^, ^29^, ^30^, ^31^, ^32^, ^33^, ^83^, ^84^ which should be given adequate attention to for the anticancer treatment in the context of COVID‐19 crisis.^8^, ^9^, ^10^, ^11^, ^12^, ^13^, ^14^, ^26^ The need of minimal invasion has already become a crucial consideration for therapeutic decision‐making in the SAUCCC.^8^, ^9^, ^10^, ^11^, ^12^, ^13^, ^14^ Accordingly, HA and HATACE possess unique superiorities among multifarious therapies for appropriate HCC patients in the SAUCCC,^17^, ^18^, ^19^, ^20^, ^22^, ^23^, ^24^, ^25^, ^26^, ^27^, ^28^, ^29^, ^30^, ^31^, ^32^, ^33^, ^85^, ^86^, ^87^, ^88^ which is fully exhibited in the Data S1.

COVID‐19 throughout the world has caused unprecedented social turmoil on a global level, triggering a comprehensive transformation of global healthcare systems.^4^, ^5^, ^6^, ^7^, ^89^, ^90^, ^91^, ^92^, ^93^, ^94^, ^95^, ^96^, ^97^ There exists dilemmatic predicament in regard to SR for HCC patients in the SAUCCC, which has been mentioned in the introduction section. So far, the minimally invasive HA and HATACE were identified as the optimal alternative to SR for applicable HCC patients in the SAUCCC.^8^, ^9^, ^10^, ^11^, ^12^, ^13^, ^14^, ^17^, ^18^, ^19^, ^20^, ^21^ The data of several meta‐analyses^17^, ^18^, ^19^, ^20^, ^21^ have already demonstrated that HA (or HATACE) offers comparable oncologic outcomes for applicable HCC patients as compared with SR and with added safety benefit of lower morbidity. (i) The specific safety advantages of HA (HATACE), such as lower incidence of complications, less intraoperative blood loss, and shorter operative time, are beneficial to reduce the risk of SARS‐CoV‐2 infection by preserving patients in a relatively good holistic state.^8^, ^9^, ^10^, ^11^, ^12^, ^13^, ^14^, ^17^, ^18^, ^19^, ^20^, ^21^ (ii) The shorter hospitalization duration, one verified superiority of HA (HATACE) compared with SR,^17^, ^18^, ^19^, ^20^, ^21^ is significant not only to minimize the risk of nosocomial cross‐infection of SARS‐CoV‐2 by reducing the exposure frequency and total duration of SARS‐CoV‐2, but also to increase the turnover rate of hospitalization. In the summer of 2021, SARS‐CoV‐2 Delta Variant surge has caused a new wave of epidemic peak in America and some other countries.^4^, ^98^ As a matter of fact, hospital beds and other medical resources have become more and more shortage due to the severely escalating COVID‐19 epidemics, causing the increasing death of both patients with COVID‐19 and without COVID‐19.^6^, ^97^, ^98^ Therefore, it is necessary and urgent to accelerate the turnover rate of hospitalization for improving the capacity of medical service and ameliorating the widespread shortage of healthcare resource in the context of the unprecedented COVID‐19 crisis.^99^, ^100^, ^101^

To our knowledge, this article is the first systematic review and meta‐analysis to evaluate HATACE for HCC, regarding ablation modalities including both RFA and MWA. Additionally, it addresses not only the largest sample size of 5036 patients from 36 included studies in this subject,^39^, ^40^, ^41^, ^42^, ^43^, ^44^, ^45^, ^46^, ^47^, ^48^, ^49^, ^50^, ^51^, ^52^, ^53^, ^54^, ^55^, ^56^, ^57^, ^58^, ^59^, ^60^, ^61^, ^62^, ^63^, ^64^, ^65^, ^66^, ^67^, ^68^, ^69^, ^70^, ^71^, ^72^, ^73^, ^74^, ^102^ but also a total of 10 RCTs for sensitivity analyses. Therefore, the present study could provide more comprehensive and reliable evidence for decision‐makings than other congeneric research.^102^ However, there are some limitations in our study indeed. The robustness of the HATACE versus HA results was demonstrated by the sensitivity meta‐analysis of RCTs with adequate qualification, but the sensitivity meta‐analysis of RCTs for HATACE versus TACE was not implemented finally because of the insufficient studies and data. So the evidential strength grade of HATACE versus TACE should be judged to be lower than that of HATACE versus HA. Nevertheless, it is very circumscribed and impractical to investigate the adverse reactions of therapies only relying on RCTs in this topic. Non‐RCT clinical studies are necessary and important for assessing the safety; therefore, the adverse reactions evaluation of HATACE, which is a key consideration for clinical decision‐making during the COVID‐19 pandemic, is relatively independent on the test strategy of meta‐analysis with RCTs. Hence, the conclusions on the safety of HATACE, including that HA significantly reduces the toxicity of TACE and preserves the liver function to some extent, could be considered adequately reliable.

In this study, we have demonstrated that HATACE for HCC is superior to TACE monotherapy with respect to either efficacy or safety. HATACE is more effective than HA monotherapy with comparable safety for non‐small‐sized (>3 cm) HCC. Compared with HATACE, HA monotherapy could provide comparable survival benefit for the patients with small (≤3 cm) HCC. Namely, adjuvant TACE is not necessary for HA therapy in treating small HCC. Although there are some deficiencies as discussed in limitations above, this research could provide a comprehensive reference for clinical decision‐making on the base of the 36 included studies and the adequately large sample size of 5036 patients. In addition, we should pay more attention to HA and HATACE due to their superiorities in the SAUCCC.

## CONFLICT OF INTEREST

The authors declare no conflict of interest. The authors alone are responsible for the content and writing of the article.

## ETHICAL DECLARATION

No ethical approval was required for the systematic review and meta‐analysis as all data originated from previously published studies.

## Supporting information

Data S1

## Data Availability

The authors confirm that they included a citation for available data in References section. The data that support the findings of this study are available from the corresponding author upon reasonable request.
