# Supplementary material for: Hyperthermia ablation combined with transarterial chemoembolization versus monotherapy for hepatocellular carcinoma: A systematic review and meta‐analysis
Source: Cancer Med. 2021 Oct 16;10(23):8432–50. doi: 10.1002/cam4.4350 (PMC8633247; doi:10.1002/cam4.4350)
Supplement: Supplementary file 1 — Data S1 [file CAM4-10-8432-s001.pdf]

# Supporting Information

## RESULTS

### Results of study search and screening

From our systematized search, a total of 3122 records were imported into the EndNote software for further identification, including 3099 records identified through traditional database searching and 23 records identified through additional sources. A total of 1787 reduplicative records were removed because of repeat included by the different databases. 1335 records after duplicates elimination were screened for eligibility by titles and abstracts at the first stage and by full-text at the second stage. A total of 36 eligible studies[39-74] for HATACE were included finally for the systematic review and meta-analysis (Figure 1).

### Characteristics of the included studies

All of the included studies[39-74] were published in SCI journals and included in the Web of Science with good quality of reports. The main features of the included trials[39-74] are presented in Table 1. There were 36 included studies in total for the systematic review and meta-analysis, which contained twenty-three studies[41, 43-46, 52, 53, 56-59, 61, 63-65, 67-74] of RFA, ten studies[40, 42, 47-51, 54, 62, 66] of MWA, and three studies[39, 55, 60] of RFA and MWA. Twenty-one[41, 42, 45, 48-52, 54, 57-62, 64, 66, 69, 71, 72, 74] of the HATACE studies were done in China, five[46, 67, 68, 70, 73] in Japan, five [43, 53, 56, 63, 65] in Korea, two [39, 47] in America, two [40, 55] in Egypt, and one[44] in Italy. The total sample size was 5036 patients, which

contained 2850 patients from China. There were 10 RCTs[40, 52, 55, 60, 61, 64, 67, 68, 71, 73], 5 CCTs[44, 58, 66, 72, 74], and 21 retrospective studies[39, 41-43, 45-51, 53, 54, 56, 57, 59, 62, 63, 65, 69, 70] (Table 1).

**Analysis of risk of bias in RCTs for the sensitivity analysis**

The method of random sequence generation was appropriate in five RCTs[40, 60, 61, 64, 67] and unclear in the other five RCTs[52, 55, 68, 71, 73]. Three RCTs[40, 61, 64] stated that allocations were concealed, while the others[52, 55, 60, 67, 68, 71, 73] did not report the allocation concealment. Seven RCTs[40, 52, 55, 67, 68, 71, 73] did not mention whether blinding of participants and personnel was used. Three RCTs[60, 61, 64] reported that they did not adopt blinding of participants and personnel, so Figure 3 marks the seats of corresponding item with red as "High risk of bias". However, blinding was not appropriate for the physicians who performed the HA or TACE procedures. Moreover, the primary measurements were all objective indicators that are independent of blinding. Therefore, the assessment item regarding blinding of participants and personnel in Figure 4 was judged as "Low risk of bias" in the end. Two RCTs[60, 61] reported that they implemented blinding of outcome assessment, while the others[40, 52, 55, 64, 67, 68, 71, 73] did not mention this item. There was none loss of follow-up in six RCTs[52, 55, 64, 67, 68, 71] with complete outcome data. One RCT[73] did not mention the item of incomplete outcome data. Existing cases of loss to follow-up were reported in three RCTs[40, 60, 61] respectively, but the data of loss to follow-up were all considered within the statistical acceptable range. Therefore, we assessed the item of incomplete outcome data as "Low risk of bias" finally in Figure 4 instead of "High risk of bias" in Figure 3. None of the included

studies[40, 52, 55, 60, 61, 64, 67, 68, 71, 73] had selective outcome reporting. Other biases were all unclear according to the extracted data from the RCTs[40, 52, 55, 60, 61, 64, 67, 68, 71, 73] for assessment of risk of bias.

## DISCUSSION

### HA and HATACE for HCC during the COVID-19 crisis

RFA and MWA, referred to as HA in this article, have the advantages of minimal invasion, high effectiveness and low toxicity[22, 23]. Therefore, RFA and MWA are widely used in clinical practice, not only for the treatment of cancer[22, 85], but also for the treatment of other diseases[22, 86]. RFA and MWA, the two ablation therapies as different subspecies of HA (hyperthermia ablation), have similar therapeutic safety and efficacy in the HCC treatment, which have already been demonstrated by sufficient evidence from RCT and meta-analysis[87]. Therefore, RFA and MWA were analyzed together with the unified concept of HA in this study, which is in conformity with several included studies for meta-analysis[39, 55, 60]. What are the advantages of HA for HCC treatment in the SAUCCC? HA could kill HCC with a forceful effectiveness[22, 23], which is beneficial to patient's holistic status by reducing the damage of cancer to the body (including immune-system)[79, 80]. More importantly, HA has unique superiorities for therapeutic safety[22, 23]. In addition to having the maximized safety and superior anti-cancer efficacy concurrently[22, 23], HA is helpful for enhancing the immune function of HCC patients as a unique advantage among multifarious therapies[24-26, 33], which is extremely important in the context of COVID-19 crisis[8-12]. Therefore, HA was further assessed for

therapeutic decision-making in the SAUCCC. Is there a better strategy of HA-based integrated therapy according to the principle of minimal invasion in the SAUCCC[8-12]? TACE is a standard minimally invasive therapy with the characteristic of reducing and controlling the toxicity of chemotherapy of normal tissues[35]. It is the first-line applied therapy worldwide for the specific patients with HCC in intermediate stage according to the Barcelona Clinic Liver Cancer (BCLC) guidelines[35, 78]. In order to explore the better strategy on the base of HA identification above in the SAUCCC, it is necessary to assess the potential combination of HA-based and TACE adjuvant therapy[34, 35, 88]. Plenty of studies[34, 35, 81, 88] indicated that the combination therapy of HA and TACE seems to be a better strategy than HA monotherapy for treating HCC. The optimization of treatment regimens should become the core of HCC management adjustments caused by the COVID-19 pandemic because it is urgently needed for the HCC patients worldwide to reduce malignant death. In addition, it is necessary to mutually learn to improve the treatment level for all countries to fight against HCC and SARS-CoV-2 in this disaster of historic proportions.

**The increase of safety weight caused by the COVID-19 crisis**

As a pivotal change, the COVID-19 crisis has significantly increased the weight of therapeutic safety in clinical decision-making[8-14, 95, 96]. Namely, safety has become the preferred consideration for decision-making in the SAUCCC as it is significantly associated with the risk of SARS-CoV-2 infection[8-14, 95, 96]. The truth is more intricate than it seems among COVID-19, cancer and its treatment[6-15, 93, 97]. Protecting patients from the infection of SARS-CoV-2 is a crucial consideration in the SAUCCC, which

generally results in giving up SR or delay in SR for some HCC patients on account of the high-risk of SARS-CoV-2 infection after SR[8-14]. But on the other hand, what is the optimal alternative to SR for reducing the risk of HCC malignant death? This dilemma about HCC in the SAUCCC could be settled easily and perfectly if there is an idealized therapy modality that only kills cancer without any toxicity for normal-tissue[8-14]. Accordingly, the minimally invasion for anticancer strategy is a realistic and feasible solution for the dilemmatic predicament[8-14]. The evaluation of therapeutic safety (or effectiveness) is completely independent of the COVID-19 pandemic, which means that the studies regarding comparisons between SR and minimally invasive therapies for HCC are always applicative for decision-makings whether they are before or after the COVID-19 outbreak. Although there is sufficient evidence[17-21], it is dilemmatic for decision-making sometimes when there is similar benefit between SR and HA (HATACE) for HCC patients before the COVID-19 pandemic. However, minimally invasive HA (HATACE) have already become the preferred strategy instead of SR in the SAUCCC due to their safety superiorities with low risk of SARS-CoV-2 infection[8-14, 17-21], which is significantly different from the real world before the COVID-19 pandemic. Briefly, SARS-CoV-2 enlarges the weight of safety superiorities of minimally invasive HA and HATACE in decision-makings[8-14, 17-21].

Nearly half cases and deaths of the world's liver cancers distribute in China[75, 76]. Both RFA and MWA of HA were developing rapidly in China during the past two decades. Now China has the advanced equipment and clinical technology of HA, especially in regard to MWA[22]. A total of 21 studies[41, 42, 45, 48-52, 54, 57-62, 64, 66, 69, 71, 72, 74]

with 2850 patients for the synthesized analyses came from China, which accounted for more than half of the 36 included studies[39-74] and 5036 total sample size. According to Chinese experiences, HA and HATACE in treating HCC could minimize harms to body (especially for the immune-system) and concurrently obtain excellent anti-cancer effectiveness, which is significant to preserve HCC patients in a relatively good systemic and immune condition for fighting against SARS-CoV-2 and cancer simultaneously in the context of COVID-19 crisis[23-33, 83, 84]. So far, the minimally invasive HA was identified as the optimal alternative to SR for small (≤3 cm) HCC while HATACE is recommended for non-small-sized (＞3 cm) HCC in the SAUCCC based on the data from comprehensive investigation[8-14, 17-21, 39-74]. We propose that personalized treatment recommendation should be addressed to minimize the risk of SARS-CoV-2 infection and cancerous malignant death synchronously along with meticulous personal protective protocols for HCC patients.
